# Mobile phone addiction and negative emotions: an empirical study among adolescents in Jiangxi Province

**DOI:** 10.3389/fpsyt.2025.1541605

**Published:** 2025-01-27

**Authors:** Jin Luo, Guanqing Cai, Xiaofang Zu, Qiming Huang, Qing Cao

**Affiliations:** Key Laboratory of Poyang Lake Wetland and Watershed Research, Ministry of Education, School of Geography and Environment, Jiangxi Normal University, Nanchang, China

**Keywords:** mobile phone addiction, negative emotions, adolescents, differences, restricted cubic spline, structural equation modeling

## Abstract

**Background:**

The relationship between mobile phone addiction and negative affect among adolescents is characterized by intricate interconnections. These factors have an impact on the emotional and psychological well-being of young people. While previous studies have provided preliminary insights into this relationship, further in-depth exploration and research is required to fully understand the complex mechanisms behind these relationships and to identify any group differences.

**Methods:**

This study collected questionnaire data from 493 adolescents aged 17-22 years old in Jiangxi Province. The data were analyzed using Pearson correlation analysis, restricted cubic spline (RCS), and structural equation modelling (SEM) with the aim of exploring the mechanisms and differences in the effects of mobile phone addiction on negative emotions of different youth groups.

**Results:**

The findings of the study indicated notable discrepancies in mobile phone addiction by gender and place of residence. Secondly, mobile phone addiction among adolescents positively drives negative emotions. The findings revealed that negative emotions triggered by mobile phone addiction were significantly higher among rural adolescents than urban adolescents; male adolescents exhibited higher levels than female adolescents in the same place of residence. Additionally, family factors, physical exercise and psychological resilience can directly or indirectly inhibit mobile phone addiction among adolescents. Finally, adolescents’ psychological resilience can significantly alleviate the negative emotions associated with mobile phone addiction. Physical exercise and family factors can reduce the negative emotional impact of mobile phone addiction by increasing psychological resilience.

**Conclusions:**

This study employs the relationship between mobile phone addiction and negative emotions in adolescents, reveals the complex mechanisms and group differences behind these relationships, and provides a fresh perspective for understanding the impact of mobile phone addiction on adolescents’ mental health, as well as a scientific basis for the development of effective interventions.

## 1 Introduction

Excessive mobile phone dependence is now a common problem among human beings, and the prevalence of mobile phone addiction is around 26.99% worldwide (1). However, the rate of mobile phone addiction among youth is higher than that of other populations, reaching as high as 30% (2–4). Notably, some studies report that the prevalence may be as high as 50% or more (5, 6). Excessive mobile phones use has detrimental impact on mental health (7, 8), interpersonal interactions (9), and sleep quality (10, 11). Among various age groups, mobile phone addiction has particularly pronounced negative effects on adolescents, potentially impairing cognitive functions and academic performance (12). Therefore, exploring the relationship between mobile phone addiction and negative emotions in adolescents, and uncovering the complex mechanisms and group differences behind these relationships, may provide new perspectives for understanding the impact of mobile phone addiction on adolescents’ mental health.

The issues related to mobile phone addiction have garnered significant attention from researchers across various disciplines, including psychology (13, 14), behavioral science (15, 16), and cognitive neuroscience (17, 18). Researchers have conducted extensive studies from their respective professional preferences, focusing primarily on areas such as the impact of mobile phone addiction, it’s driving factors, intervention strategies (19–21). Among these, the relationship between mobile phone addiction and mental health is a central research focus. Numerous studies have shown that mobile phone addiction can contribute to mental health issues such as depression, anxiety, and loneliness (22–25). Another key area of research is the effect of mobile phone addiction on individual behavior. Studies have indicated that excessive reliance on smartphones can disrupt learning (26, 27), work (28), and social activities (29), leading to distraction and decreased productivity. It can also result in physical problems such as joint pain (30). In adolescent mobile phone addiction has been linked to eating disorders (31), suicidal ideation (32), and lower emotional intelligence (33).

Most previous studies have shown that not only family environment (34), physical exercise (35, 36) and personal psychological resilience (37) are strongly related to mobile phone addiction and negative affect, but also that differences in residential environment and gender play a significant role in leading to differences in mobile phone addiction and negative affect (5). In particular, a good family environment has a significant inhibitory effect on mobile phone addiction and negative affect (38), in terms of family cultural capital, family economic capital and family social capital. In families with a high level of cultural capital, adolescents are less likely to exhibit addictive behavior with regard to mobile phones and to experience negative emotions. This is because the family’s values, which emphasize the importance of education and cultural activities, can guide adolescents to invest more time and energy into learning and other positive activities (39, 40). And families rich in economic capital can provide adolescents with more resources and opportunities, which can help reduce adolescents’ dependence on mobile phones (41, 42). Family social capital includes the quality of social interactions and relationships between family members. High quality parent-child relationships and a harmonious family atmosphere can significantly reduce adolescents’ mobile phone dependence and negative emotions (43).

The propensity for physical exercise and psychological resilience are significant factors in the alleviation of negative emotions. Firstly, it has been demonstrated that physical exercise has an effective enhancing effect on an individual’s physical health, thereby contributing to a reduction in negative emotions (44). Moreover, it is beneficial for enhancing an individual’s sense of self-efficacy and fortifying their capacity to navigate challenges, thereby mitigating negative emotions (45). Moreover, evidence indicates that physical exercise can facilitate the development of social interaction skills and reinforce an individual’s social support network, which has been linked to a reduction in negative emotions. Secondly, individuals who possess robust psychological resilience are better equipped to navigate challenges in life, which in turn reduces negative emotions. Moreover, psychological resilience allows individuals to adapt more effectively to environmental changes, which in turn reduces negative emotions.

Furthermore, discrepancies in living circumstances and gender may also be implicated in the development of mobile phone addiction and the manifestation of negative mood states. A review of the literature reveals that women are more likely to experience problems with mobile phone use than men (46). This may be attributed to the observation that women are more prone to mood swings, which may lead them to use their mobile phones as a means of detoxifying negative emotions (47). Residing in a rural setting may engender feelings of solitude and detachment, whereas urban living may precipitate heightened stress and anxiety, which may in turn precipitate mobile phone addiction (48, 49). Additionally, the potential moderating effects of gender and place of residence had a significant impact on the relationship between loneliness and smartphone addiction (50), indicating a pivotal role for the interaction of gender and residential environment in the development of mobile phone addiction. Those experiencing negative emotions are more likely to engage in problematic mobile phone use as a means of excreting these emotions, thereby further exacerbating the problem of mobile phone addiction (51, 52). These factors contribute to the development of mobile phone addiction and may exacerbate the associated negative emotions by influencing an individual’s mental state, behavioral patterns, and emotional regulation.

However, although existing studies have developed preliminary insights into the relationship between mobile phone addiction and negative emotions in adolescents, they have not comprehensively explored the complex mechanisms and group differences behind mobile phone addiction and negative emotions (53). Furthermore, psychological resilience, a crucial factor in the relationship between mobile phone addiction and negative emotions, has been largely overlooked in the majority of studies. Finally, the inclusion of additional factors, such as family environment, physical exercise, psychological resilience, residential environment, and gender differences, is essential for a more comprehensive and objective exploration of the effects of mobile phone addiction on negative emotions.

This study collected questionnaire data from 493 adolescents aged 17-22 from Jiangxi Province with the objective of exploring the differences in mobile phone addiction among different youth groups. Subsequently, Pearson correlation analysis and restricted cubic spline were employed to quantify the effects of differences in mobile phone addiction on negative emotions among different groups. Finally, structural equation modelling was utilized to explore the effects of mobile phone addiction on negative emotions of different youth groups through the mechanisms and differences.

## 2 Materials and methodology

### 2.1 Study area

Jiangxi Province, located in the southeastern part of the People’s Republic of China, is bordered to the south by the middle and lower reaches of the Yangtze River basin. The province’s geographical coordinates range from 24°29′14″ to 30°04′43″ north latitude and from 113°34′18″ to 118°28′56″ east longitude. It is comprised of 11 prefecture-level cities and covers an area of 166,900 square kilometers. As of 2023, the estimated resident population is 45,150,100.

### 2.2 The collection of questionnaire data

This study focused on a sample of 493 adolescents from Jiangxi Province, China, in 2024, with ages ranging from 17 to 22 years. The sample included 55 urban males, 165 urban females, 59 rural males, and 214 rural females. This age group was chosen because they are in the transition from adolescence to adulthood, and their psychological and behavioral patterns are in a period of rapid development and change, making them more susceptible to the effects of mobile phone addiction. A previous meta-analysis of mobile phone addiction in children and adolescents by Sohn et al. (54) found that adolescents in the 17-19 age group exhibited a higher prevalence of mobile phone addiction. Additionally, adolescents aged 17 - 22 are facing the transition from high school to college or from school to the workplace, and the change in environment and roles may lead to adjustment problems and psychological distress. Therefore, it is important to study this specific age group to reveal the impact of mobile phone addiction on negative emotions.

Questionnaires were used to collect data on the adolescents’ mobile phone addiction behaviors, negative emotional states, psychological resilience, and basic information regarding their familial and educational backgrounds. The survey covered the following specific areas:

#### 2.2.1 Fundamental information survey of individuals, families, and schools

The personal information collected includes gender, age, household registration type (urban or rural), high school of graduation, and daily duration of physical exercise. For convenience of subsequent analysis, gender is coded as 1 for “male” and 2 for “female”; household registration type is coded as 1 for “urban household registration” and 2 for “rural household registration.” Additionally, the geographic coordinates of the adolescents’ high schools were obtained using the Gaode Map service. The duration of physical exercise is considered a potential variable that may influence negative emotions.

The family information collected includes the parents’ occupations, educational levels, family property, number of books, and tutoring fees during the senior year of high school. Parents’ occupations are scored on a scale from 1 to 12, with 12 representing the highest social prestige and 1 representing the lowest, based on 12 categories. Educational levels are scored from 1 (primary school education) to 7 (doctoral education), with 7 options in total. Family property is assessed across 17 categories, with each category assigned 1 point, and the total family property score reflects the number of categories owned. The number of family books is evaluated on a 1-6 point scale, with higher numbers of books corresponding to higher scores. Tutoring fees during the senior year are assessed across 5 fee range options, using a 5-point system, where higher fees correspond to higher scores. The family’s social capital is measured by the average score of parents’ occupational prestige, while cultural capital is represented by the average score of parents’ educational levels. The family’s economic capital is calculated by summing the scores for family property, number of books, and tutoring fees during the senior year.

#### 2.2.2 Evaluation of mobile phone addiction

The current study employed the Smartphone Addiction Scale - Short Version (SAS-SV) to assess mobile phone addiction in adolescents (55, 56). This scale has been widely used by Chinese researchers to investigate and study mobile phone addiction among Chinese adolescents, with high reliability (57–59). The SAS-SV consists of 10 behavioral statements related to mobile phone addiction, with each statement rated on a 5-point scale (1 =“strongly disagree” 5 =“strongly agree”). The mean score of these 10 statements was used to determine the severity of the adolescent’s mobile phone addiction, with higher score indicating more severe problem.

#### 2.2.3 Evaluation of negative emotions

The Depression Anxiety Stress Scales-21 (DASS-21) was used to assess negative emotional states in adolescents (60). The validity of the scale has been well established in different Chinese populations (61), such as Zhang (2024), who found that the DASS-21 exhibited strict invariance between genders and weak invariance across education levels by surveying 7943 Chinese students (62). The DASS-21 consists of 21 items that measure negative emotions across three sub-dimensions: anxiety (items 1–7), depression (items 8–14), and stress (items 15–21). Each item is rated on a five-point Likert scale, with 1 indicating strong disagreement and 5 indicating strong agreement. Higher average scores across the 21 items are indicative of greater negative emotional distress in adolescents.

#### 2.2.4 Evaluation of psychological resilience

This study uses the Connor-Davidson Resilience Scale (CD-RISC) to assess an individual’s capacity for coping and adaptation in the face of adversity, hardships, or traumatic events, referred to as resilience (63). Which has been empirically validated for use in psychological resilience assessments of Chinese adolescents (64, 65). The CD-RISC consists of 25 items, each with five levels of response options, rated on a 5-point Likert scale (1 = “almost never” to 5 = “almost always”). The average score across the 25 items is calculated, with higher scores indicating stronger resilience in adolescents.

Following the detailed analysis and processing of the collected questionnaire data, several indicators were derived, including mobile phone addiction, negative emotions, psychological resilience, exercise duration, and family factors. Negative emotions are assessed across three dimensions: depression, anxiety, and stress. Family factors are divided into three sub-dimensions: family cultural capital, family social capital, and family economic capital.

### 2.3 Methodology

#### 2.3.1 Pearson correlation analysis

Pearson correlation analysis is a statistical method used to assess the strength and direction of a linear relationship between two continuous variables (66). In this study, Pearson correlation analysis was applied to examine the relationships among variables such as mobile phone addiction and negative emotions in a sample of adolescents from diverse urban and rural backgrounds and genders.

#### 2.3.2 Restricted cubic splines

RCS is a statistical method used for regression analysis and curve fitting (67). This technique involves partitioning the data range of a continuous variable and fitting a cubic polynomial to each segment, thereby creating a smooth curve. In this study, the RCS method was employed to further investigate the relationship between mobile phone addiction and negative emotions in adolescents, to explore how varying level of mobile phone addiction contribute to the generation of negative emotions.

#### 2.3.3 Structural equation modeling

SEM is a statistical method used to elucidate the complex interrelationships among observed and latent variables (68). It is widely applied across various disciplines, including sociology, management, and medicine (69). It can simultaneously handle the relationships among multiple variables and clearly present their direct and indirect effect paths. For these reasons, this study employs SEM to examine the complex effects of mobile phone addiction on negative emotions, as well as the moderating roles of psychological resilience, physical exercise, and family factors in this relationship.

## 3 Result

### 3.1 Descriptive statistics and tests of difference

In this study, descriptive statistical analysis was conducted on the data from 493 adolescent samples using SPSS 26.0 software. The results of the analysis are presented in **Table 1**. The mobile phone addiction scores ranged from a minimum of 1 to a maximum of 5, with an average score of 2.92. The negative emotion scores ranged from 1 to 4.1, with an average score of 2.02.

**Table 1:** Descriptive statistics of variables.

Variable	Number	Mean	Maximum	Minimum	Standard Deviation
Negative emotions	493	2.02	4.1	1	0.67
Mobile phone addiction	493	2.92	5	1	0.74
Psychological resilience	493	3.32	5	1	0.57
Exercise intensity	493	0.5	1	0	0.5
Family cultural capital	493	4.6	12	2	1.88
Family social capital	493	12.12	24	2	4.65
Family economic capital	493	12.34	27	3	4.52
Depression	493	1.91	4.71	1	0.7
Anxiety	493	1.98	4	1	0.69
Pressure	493	2.17	4.86	1	0.83

**Figure 1:**
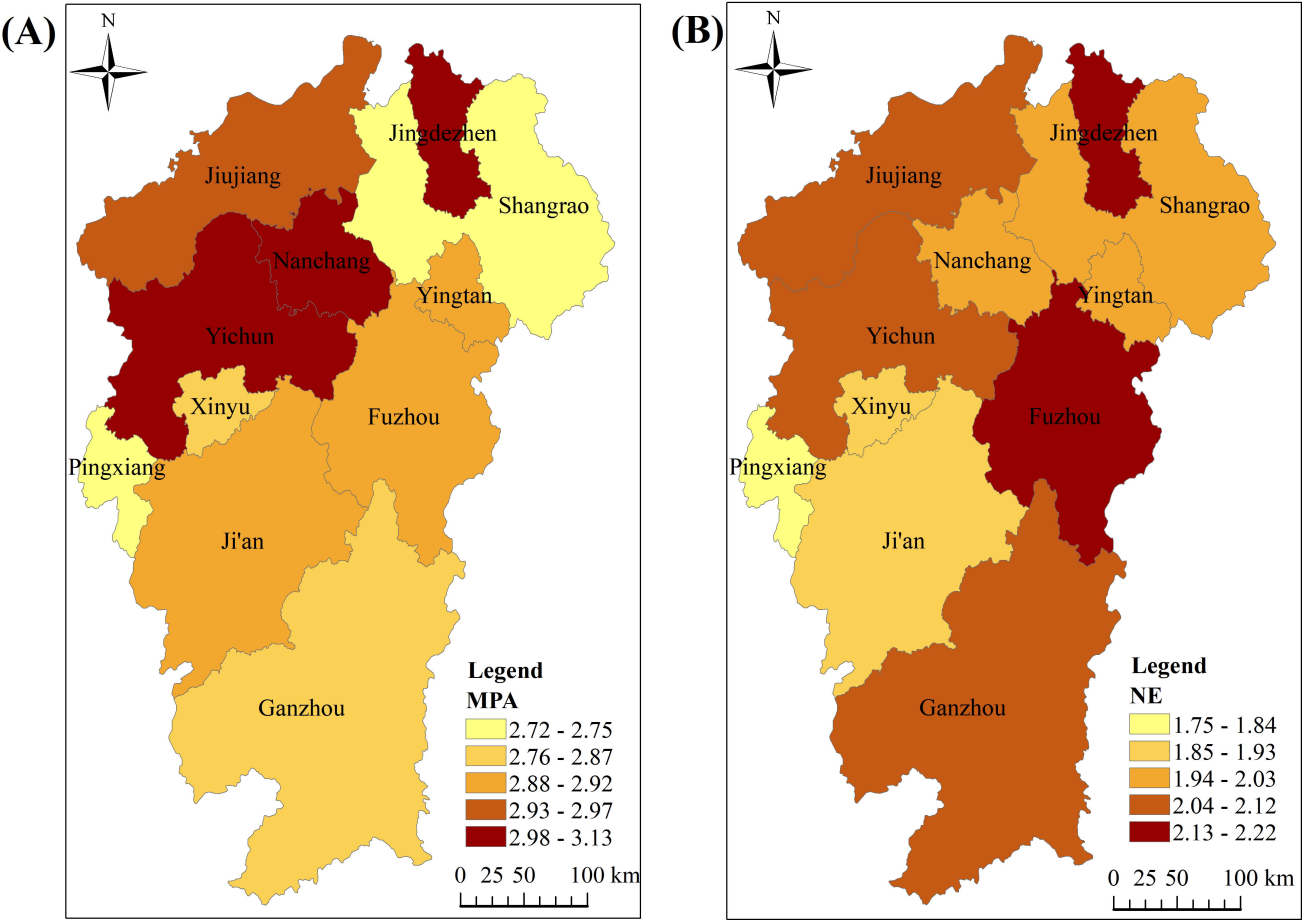
Mean distribution of mobile phone addiction **(A)** and negative emotions **(B)** among adolescents.

**Figure 1** presents the distribution of mean scores for mobile phone addiction (Panel A) and negative emotions (Panel B) across samples from various prefecture-level cities. Panel A shows that adolescents in the northern region of Jiangxi exhibit relatively higher mobile phone addiction scores, particularly in the cities of Jingdezhen, Nanchang, and Yichun. In contrast, the cities of Pingxiang and Shangrao report lower mobile phone addiction scores among their adolescent populations. Panel B highlights that adolescents in Jingdezhen and Fuzhou have elevated negative emotion scores, while those in Pingxiang exhibit the lowest negative emotion scores. A comprehensive analysis revealed that adolescents in Jingdezhen demonstrated high scores on both mobile phone addiction and negative emotions, while adolescents in Pingxiang had the lowest scores on both dimensions.

**Figure 2:**
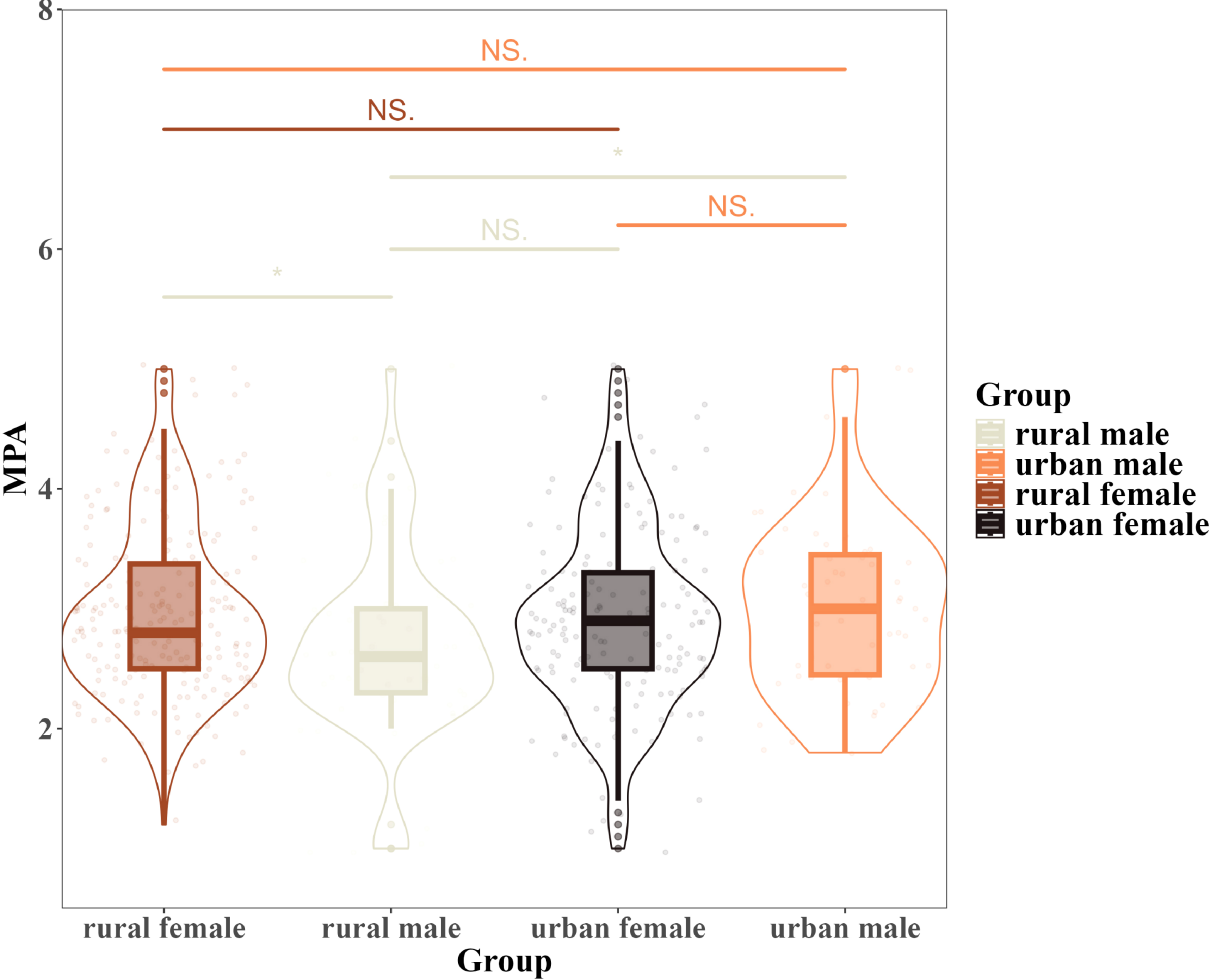
Box plots with violins for mobile phone addiction in four samples and significance test for difference between groups. “NS” stands for ‘not significant’ and indicates that the difference in mobile phone addiction among adolescents between the two groups is not significant; “*” indicates that the difference in mobile phone addiction among adolescents between the two groups is significant.

To account for differences based on urban-rural background and gender, this study divided the sample into four subgroups: urban males, urban females, rural males, and rural females. The between-group variability in mobile phone addiction and negative emotions was then examined. The findings revealed no significant differences in negative emotions across the four subgroups. However, notable discrepancies were observed in mobile phone addiction (**Figure 2**). In **Figure 2**, a horizontal line connecting two groups indicates a comparison of their differences. An asterisk (*) denotes a statistically significant difference, while “NS” indicates no significant difference. As shown in **Figure 2**, a significant discrepancy is evident between urban males and rural males regarding mobile phone addiction scores. A similar pattern is observed when comparing rural males and rural females.

### 3.2 Differential analysis of factors influencing negative emotions

**Figure 3:**
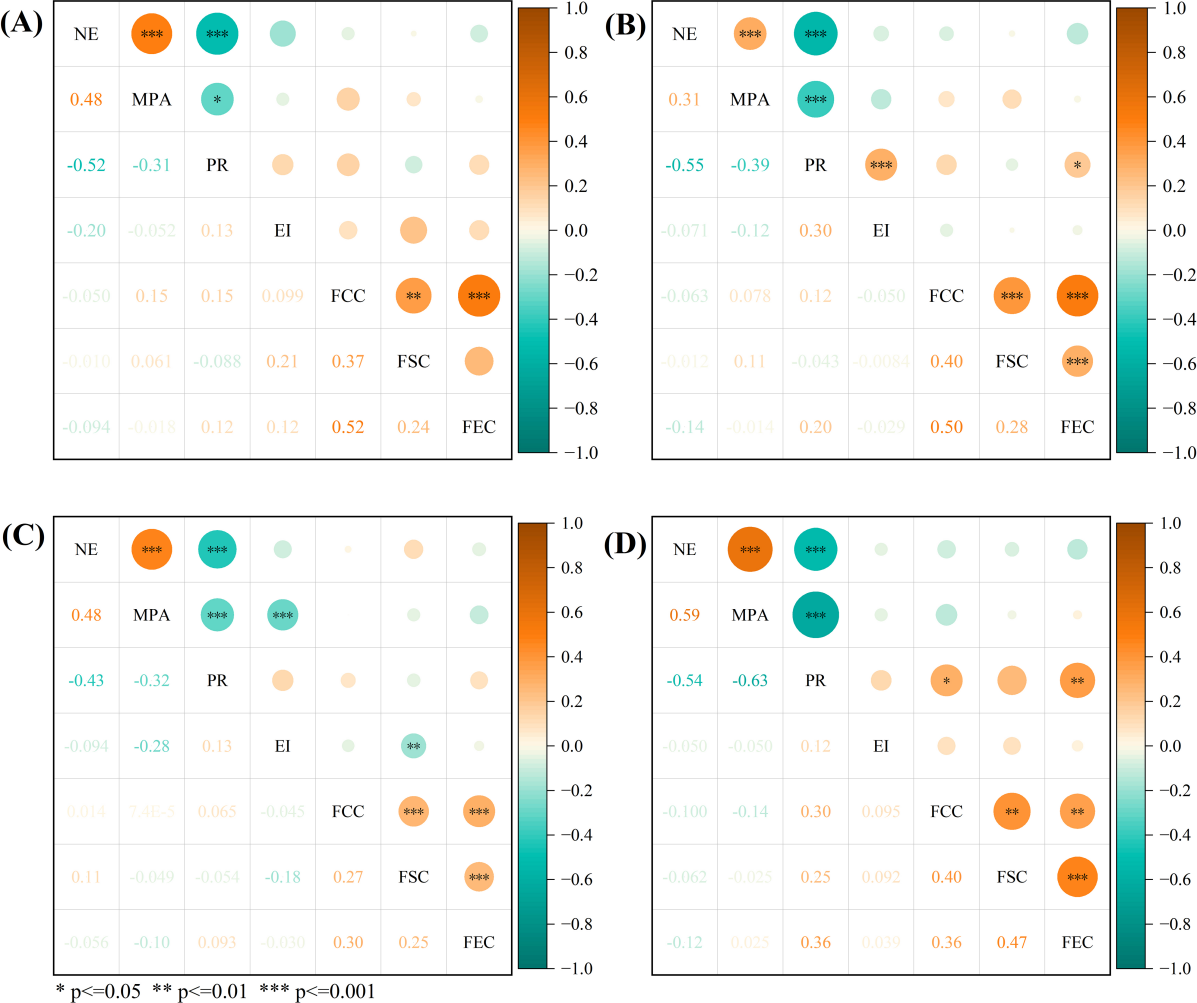
Presents the heatmap of the correlation between mobile phone addiction, negative emotions, and other factors across four adolescent groups: **(A)** urban male adolescents, **(B)** urban female adolescents, **(C)** rural male adolescents, and **(D)** rural female adolescents. The abbreviations used in the figure are as follows: “NE” stands for negative emotions, “MPA” for mobile phone addiction, “PR” for psychological resilience, “EI” for exercise intensity, “FCC” for family cultural capital, “FSC” for family social capital, and “FEC” for family economic capital. “*” indicates a significance level of p<=0.05, which means that the correlation between the two variables is generally significant; ‘**’ indicates p<=0.01, which is relatively significant; “***” indicates p<=0.001, which is highly significant.

In order to investigate the impact of differences in mobile phone addiction on negative emotions, this study employed Pearson correlation analysis to examine the relationship between variables, including mobile phone addiction and negative emotions, with particular attention to urban-rural and gender differences. The resulting correlation heatmap is shown in **Figure 3**. As depicted, a significant positive correlation exists between mobile phone addiction and negative emotions for both male and female adolescents in both urban and rural settings. Specifically, the correlation coefficients were r = 0.48 (p < 0.001) for males and r = 0.31 (p < 0.001) for females in urban areas, and r = 0.48 (p < 0.001) for males and r = 0.59 (p < 0.001) for females in rural areas.

A significant negative correlation was found between psychological resilience and both mobile phone addiction and negative emotions. The correlation coefficients between psychological resilience and mobile phone addiction for the four adolescent groups were as follows: for urban adolescents, r = -0.31 (p < 0.05) for males and r = -0.39 (p < 0.001) for females; for rural adolescents, r = -0.32 (p < 0.001) for males and r = -0.63 (p < 0.001) for females. The correlation coefficients between psychological resilience and negative emotions were as follows: for urban adolescents, r = -0.52 (p < 0.001) for males and r = -0.55 (p < 0.001) for females; for rural adolescents, r = -0.43 (p < 0.001) for males and r = -0.54 (p < 0.001) for females.

Further analysis revealed that the psychological resilience of urban female adolescents was significantly positively correlated with exercise intensity (r = 0.3, p < 0.001) and family economic capital (r = 0.2, p < 0.05). Additionally, the psychological resilience of rural female adolescents showed a significant positive correlation with family cultural capital (r = 0.3, p < 0.05) and family economic capital (r = 0.36, p < 0.01).

### 3.3 Impact of mobile phone addiction differences on negative emotions

**Figure 4:**
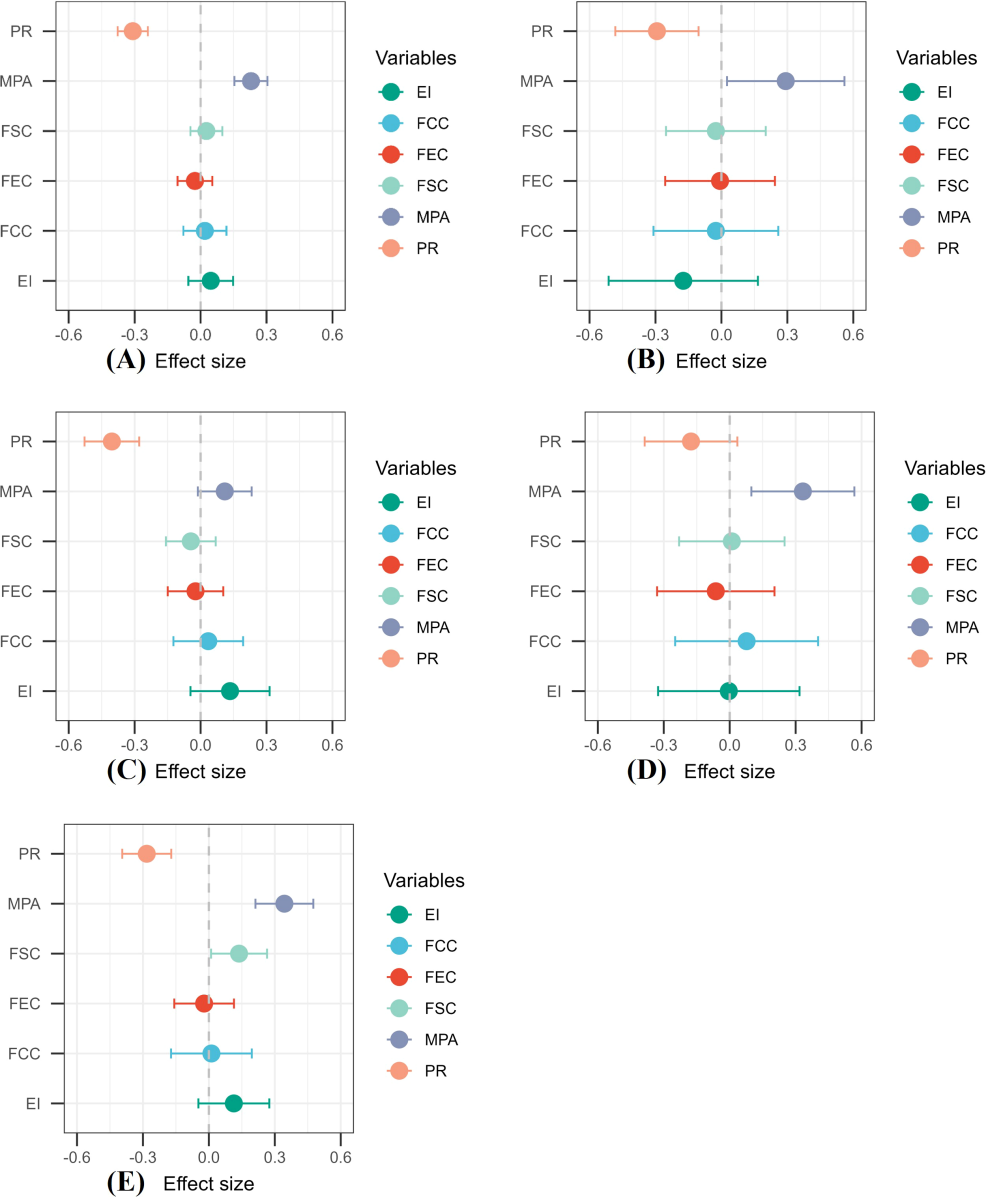
Forest plot of factors influencing negative emotions for the overall and four sample groups **(A)** overall; **(B)** urban males; **(C)** urban females; **(D)** rural males; **(E)** rural females. PR, psychological resilience; MPA, mobile phone addiction; FSC, family social capital; FEC, family economic capital; FCC, family cultural capital; EI, exercise intensity.

**Figure 5:**
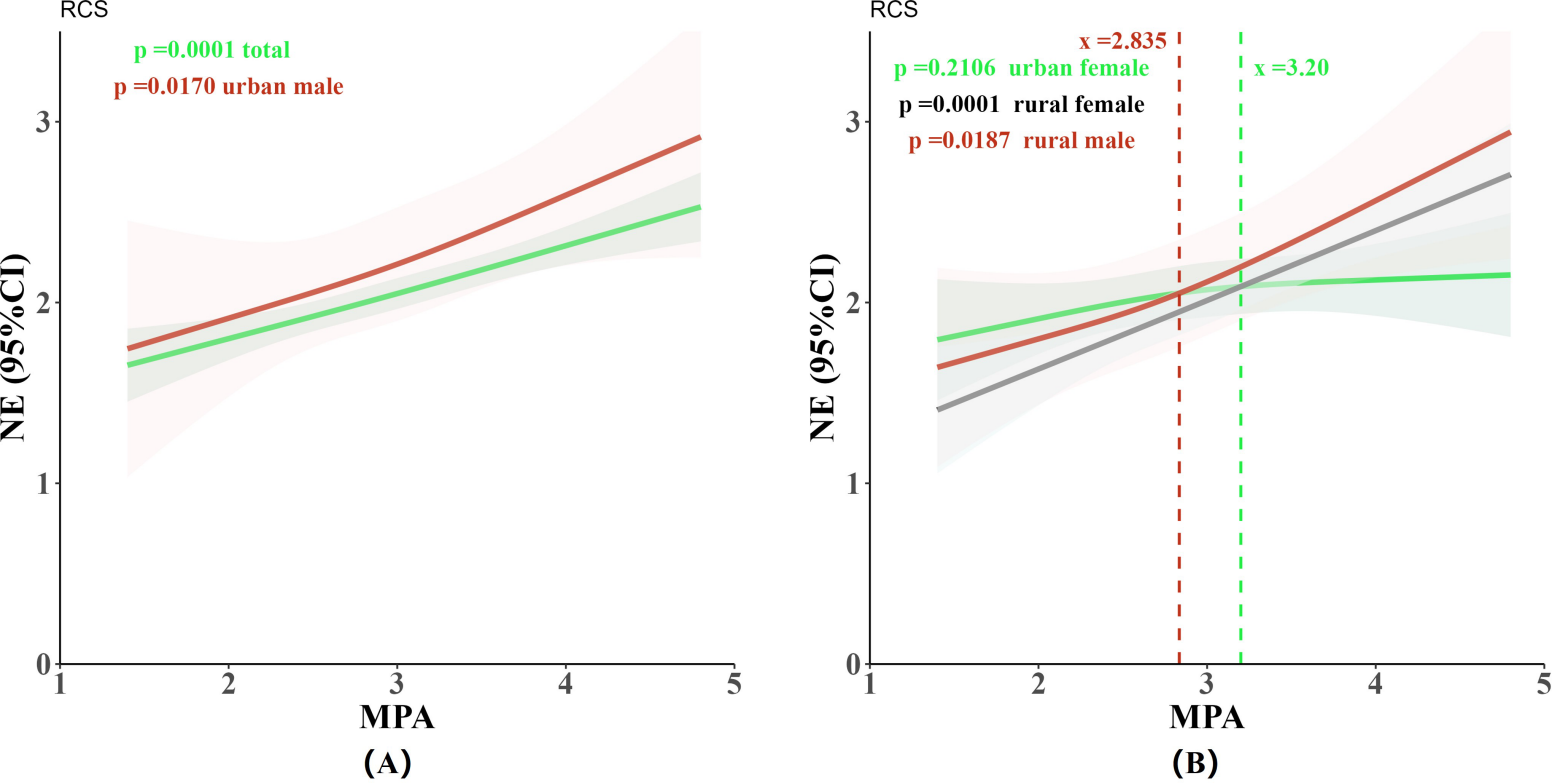
RCS plots illustrating the relationship between mobile phone addiction and negative emotions among adolescents, for the overall sample and by subgroup: **(A)** Overall sample (green) and urban males (red). **(B)** Urban females (green), rural males (red), and rural females (gray).

To further explore the impact of mobile phone addiction differences on negative emotions among different groups of adolescents, we used the RCS to quantify the relationship between the two. The overall sample was divided into four subgroups based on urban/rural and gender characteristics. The relationship between mobile phone addiction and negative emotions was then investigated using RCS analysis, both at the overall level and within each subgroup. Additionally, the variability of this relationship across the subgroups was examined. **Figure 4** presents a forest plot showing the impact of each driving factor on negative emotions for the overall sample and the four subgroups. **Figures 5A, B** displays the effect curves illustrating the relationship between mobile phone addiction and negative emotions for the overall sample as well as each subgroup. Specifically, the three fitted curves in **Figure 5A** depict the mobile phone addiction-negative emotions relationship for urban females, rural females, and rural males, highlighting the differences across these groups. **Figure 5B** shows the RCS plots for the overall sample and urban males.

As illustrated in **Figure 4**, mobile phone addiction generally has a positive effect on negative emotions (β = 0.229, 95% CI: 0.154–0.305). In contrast, psychological resilience negatively impacts negative emotions (β = -0.309, 95% CI: -0.377 to -0.240). However, the effects of physical exercise intensity, family cultural capital, family social capital, and family economic capital on negative emotions are not statistically significant. Among the four sample groups, an increase in mobile phone addiction is associated with heightened negative emotions in urban males (β = 0.293, 95% CI: 0.025–0.560), rural males (β = 0.333, 95% CI: 0.099–0.567), and rural females (β = 0.344, 95% CI: 0.212–0.475), but no such effect is observed in urban females. Conversely, as psychological resilience increases, negative emotions decrease across all groups: urban males (β = -0.293, 95% CI: -0.483 to -0.104), urban females (β = -0.404, 95% CI: -0.528 to -0.279), rural males (β = -0.176, 95% CI: -0.387 to 0.035), and rural females (β = -0.283, 95% CI: -0.394 to -0.171). The effects of physical exercise, family cultural capital, and family economic capital on negative emotions remain statistically insignificant. Additionally, family social capital is found to positively predict negative emotions in rural females (β = 0.137, 95% CI: 0.010–0.265), though this effect is not significant in the other three groups.

As illustrated in **Figure 5**, a linear relationship between mobile phone addiction and negative emotions was observed among the 493 adolescents (p overall < 0.001, p non-linear > 0.05). This relationship indicates that as the level of mobile phone addiction increased, adolescents experienced a corresponding rise in negative emotions. Additionally, a linear correlation was found in urban males (p overall < 0.05, p non-linear > 0.05). In contrast, the negative emotions of urban females showed a slower rate of increase in relation to mobile phone addiction. However, no statistically significant correlation was observed between mobile phone addiction and negative emotions in urban females (p overall > 0.05, p non-linear > 0.05). Both rural males and rural females exhibited a linear relationship between mobile phone addiction and negative emotions (rural males: p overall < 0.05, p non-linear > 0.05; rural females: p overall < 0.001, p non-linear > 0.05), suggesting that as mobile phone addiction increases among rural adolescents, their negative emotions also intensify.

Furthermore, a notable disparity was observed in the response of negative emotions related to mobile phone addiction between urban and rural females. When the mobile phone addiction score is below 3.2, urban females exhibit a higher risk of negative emotions compared to rural females. However, when the score exceeds 3.2, rural females show a greater prevalence of negative emotions associated with mobile phone addiction than their urban counterparts. This suggests that rural females are more vulnerable to negative emotions when mobile phone addiction reaches a severe level. Additionally, the curve depicting the relationship between mobile phone addiction and negative emotions among rural male adolescents (red curve) consistently shows higher levels of negative emotions than that of rural female adolescents (dark gray curve). This indicates that rural male adolescents experience more intense negative emotions than rural female adolescents at the same level of mobile phone addiction.

### 3.4 Results of structural equation modelling of the influence of mobile phone addiction and other factors on negative emotions

#### 3.4.1 Performance of structural equation modeling

**Figure 6:**
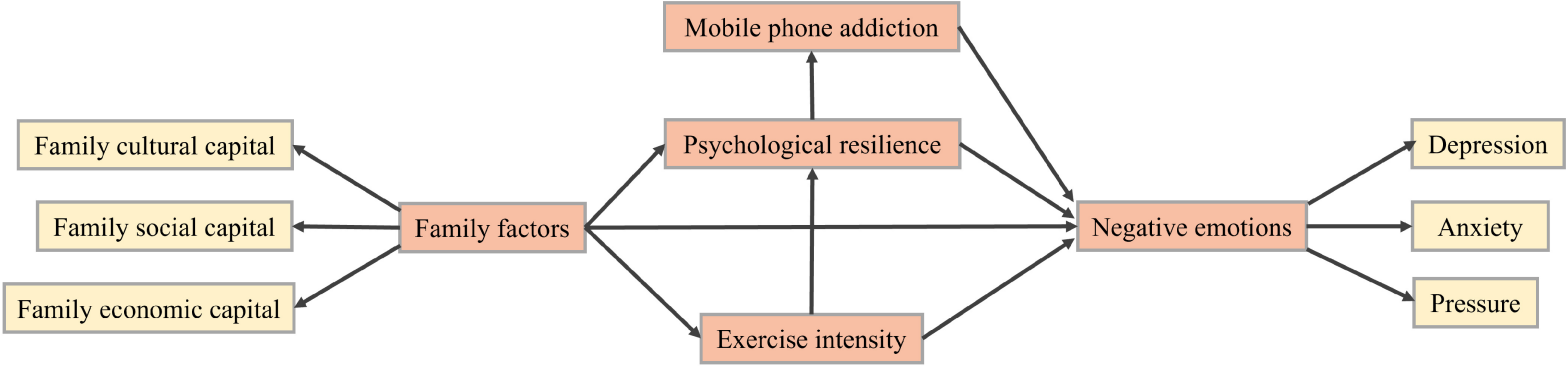
Hypothetical model of structural equations for mobile phone addiction, negative emotions.

In the preceding analysis, significant disparities in mobile phone addiction were observed among adolescents from various urban and rural backgrounds, as well as between different genders. Although the RCS is capable of quantifying the relationship between mobile phone addiction and negative emotions, it has not yet been able to explain the common effect of multiple variables, including mobile phone addiction, on negative emotions. Accordingly, the study will investigate the underlying mechanisms and the discrepancies in the impact of mobile phone addiction on negative emotions across distinct adolescents through the utilization of SEM. **Figure 6** illustrates the SEM for the four groups, which was iteratively tested and refined. In this model, negative emotion, mobile phone addiction, psychological resilience, daily exercise duration, and family factors were designated as latent variables. Depression, anxiety, and stress were treated as observed variables within the broader category of negative emotions. Although significant correlations were observed between family cultural capital, family social capital, and family economic capital in earlier correlation analyses, this study did not explore their interactions in depth. Instead, these factors were included in the model as observed variables under the umbrella of family factors.

**Table 2** presents the performance statistics of the SEM for the four data sets. According to the data in **Table 2**, the SEM for all four sample sets demonstrate excellent model fit. Specifically, the ratio of chi-square to degrees of freedom (chisq/df) is less than 3, indicating an acceptable level of model adequacy. Moreover, the Goodness of Fit Index (GFI) for urban males, urban females, rural males, and rural females is greater than or close to 0.9, the Comparative Fit Index (CFI) exceeds 0.9, the Standardized Root Mean Square Residual (SRMR) is below 0.08, and the Root Mean Square Error of Approximation (RMSEA) is below 0.1. The favorable performance of these indices confirms that all four sets of SEM have passed the fitness tests, validating the robustness and reliability of the models.

**Table 2:** Parameters of the SEM for the four sample data sets.

Group	chisq	df	chisq/df	GFI	CFI	RMR	SRMR	RMSEA
Urban male	32.928	22	1.50	0.887	0.933	0.145	0.067	0.095
Urban female	28.708	22	1.30	0.964	0.986	0.14	0.048	0.043
Rural male	32.96	22	1.50	0.887	0.949	0.207	0.069	0.092
Rural female	60.474	22	2.75	0.944	0.927	0.111	0.066	0.09

#### 3.4.2 Path coefficient analysis

**Figure 7:**
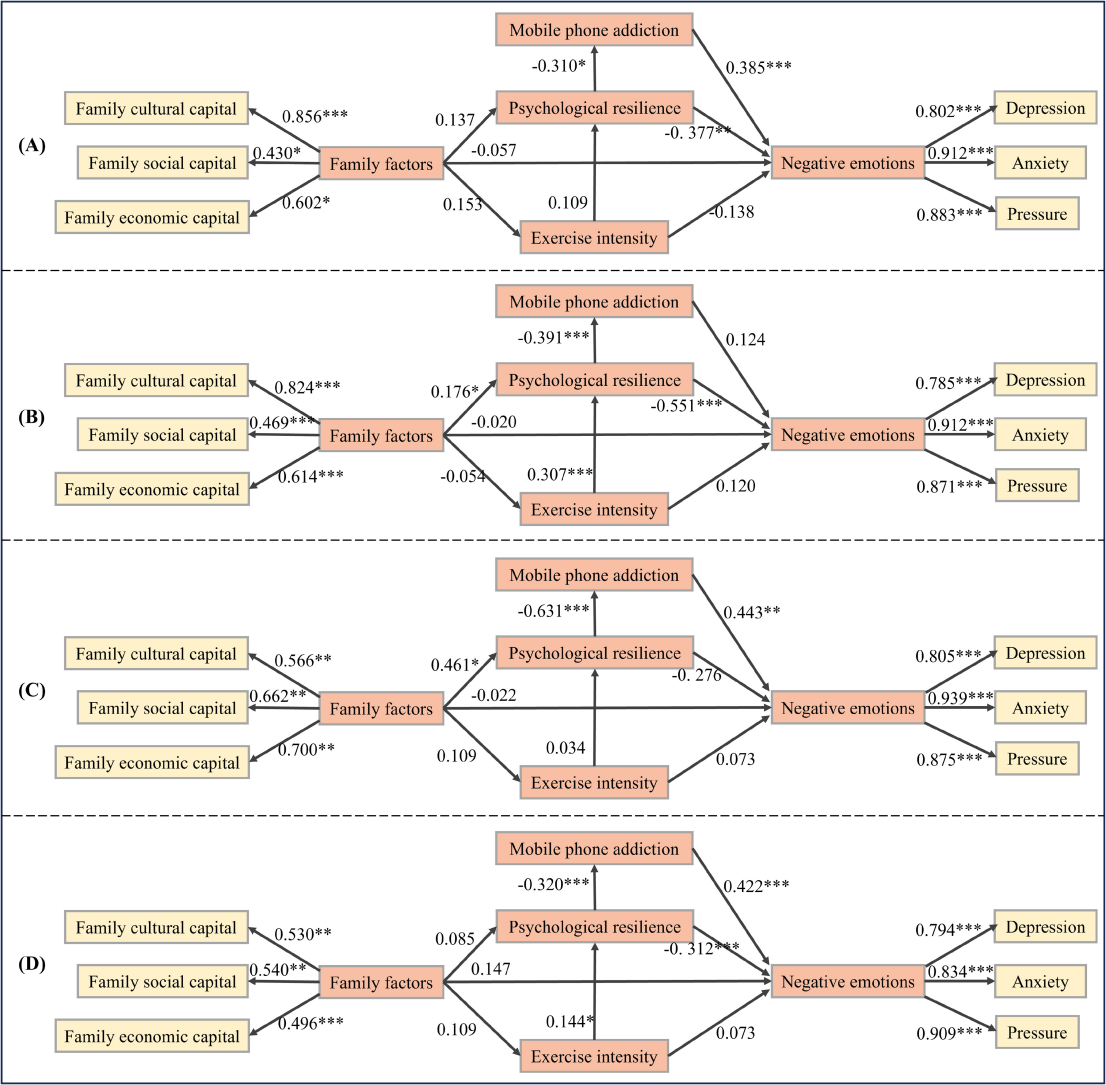
Structural Equation Path Coefficient Plots for Four Sample Data Sets **(A)** Urban males; **(B)** Urban females; **(C)** Rural males; **(D)** Rural females. “*” indicates a significance level of p<=0.05, which means that the correlation between the two variables is generally significant; “**” indicates p<=0.01, which is relatively significant; “***” indicates p<=0.001, which is highly significant.

**Figure 7** presents the path coefficient plots for the SEM across the four data sets. The path coefficients for each relationship, along with their corresponding significance levels, are clearly labeled.

As shown in **Figure 7**, among urban male adolescents, mobile phone addiction was positively associated with negative affect (β = 0.385, p < 0.001). In contrast, psychological resilience showed a significant negative correlation with negative affect (β = -0.377, p < 0.01) and also negatively correlated with mobile phone addiction (β = -0.31, p < 0.05). Although family factors (β = -0.057, p > 0.05) and exercise duration (β = -0.138, p > 0.05) exerted a negative influence on negative affect, these effects were not statistically significant.

In contrast to urban male adolescents, urban female adolescents did not exhibit a significant relationship between mobile phone addiction and negative emotions (β = 0.124, p > 0.05). However, psychological resilience had a significant negative effect on both negative emotions (β = -0.551, p < 0.001) and mobile phone addiction (β = -0.391, p < 0.001). Additionally, both exercise duration (β = 0.307, p < 0.001) and family factors (β = 0.176, p < 0.05) were positively associated with psychological resilience.

Among rural male adolescents, mobile phone addiction had a significant positive effect on negative emotions (β = 0.443, p < 0.001). While psychological resilience did not significantly affect negative emotions (β = -0.276, p > 0.05), it had a strong negative effect on mobile phone addiction (β = -0.631, p < 0.001). Family factors showed no significant effect on negative emotions (β = -0.022, p > 0.05), but had a significant positive effect on psychological resilience (β = 0.461, p < 0.05). Additionally, exercise duration did not significantly impact negative emotions (β = 0.034, p > 0.05).

Among rural female adolescents, mobile phone addiction had a significant positive effect on negative emotions (β = 0.422, p < 0.001). Psychological resilience was found to have a significant negative effect on both mobile phone addiction (β = -0.32, p < 0.001) and negative emotions (β = -0.312, p < 0.001). Exercise duration significantly positively influenced psychological resilience (β = 0.144, p < 0.05). However, family factors did not have a significant effect on negative emotions (β = 0.147, p > 0.05).

#### 3.4.3 Analysis of direct and indirect impact effects

**Figure 8:**
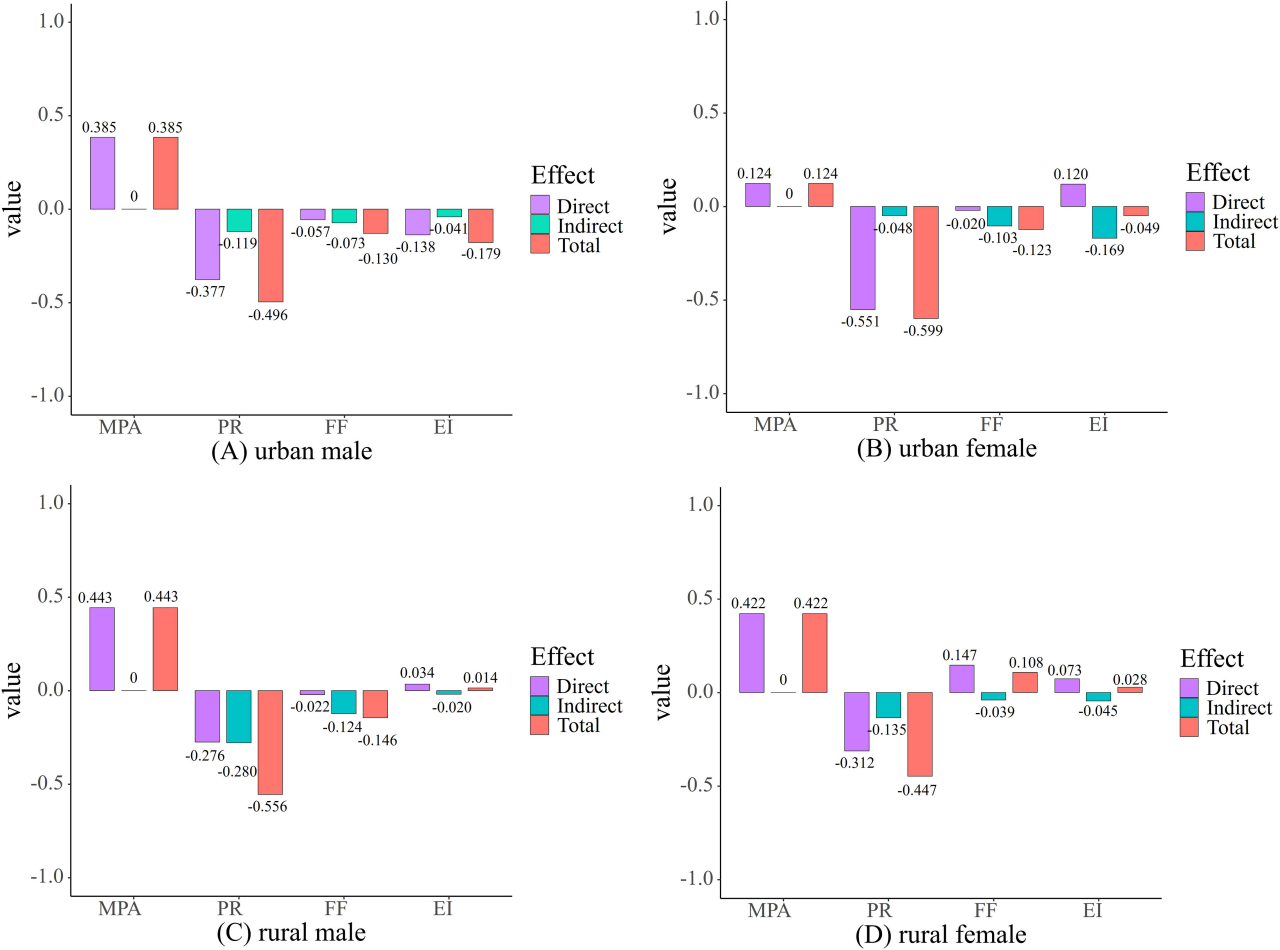
Direct, indirect and total effects of variables on negative emotions: **(A)** urban male; **(B)** urban female; **(C)** rural male; **(D)** rural female. MPA, mobile phone addiction; PR, psychological resilience; FF, family factors; EI, exercise intensity.

**Figure 8**: The Influence of Each Variable on Negative Emotional States. As illustrated in **Figure 8**, the effects of the variables on negative emotions can be summarized as follows:

In the four groups of adolescent samples, the effect of mobile phone addiction on negative emotions is exclusively direct, and the magnitude of this effect varies across the groups. The degree of influence follows the order: Rural Males (β = 0.443)> Rural Females (β = 0.422)> Urban Males (β = 0.385)> Urban Females (β = 0.124).

Among all the variables examined, psychological resilience had the most significant total effect on negative emotions. The total effect values were as follows: urban males (β = -0.496), urban females (β = -0.599), rural males (β = -0.556), and rural females (β = -0.447). The rank order of the direct effect of psychological resilience on negative emotions was urban females (β = -0.551) > urban males (β = -0.377) > rural females (β = -0.312) > rural males (β = -0.276). In contrast, the indirect effects of psychological resilience on negative emotions, mediated by mobile phone addiction, followed this rank order: rural males (β = -0.280) > rural females (β = -0.135) > urban males (β = -0.119) > urban females (β = -0.048). It is noteworthy that the direct effect of psychological resilience on negative emotions was substantially higher in urban females (β = -0.551) compared to their indirect effect (β = -0.048), with the direct effect being significantly greater than in the other three groups. Furthermore, while rural males showed the lowest direct effect of psychological resilience on negative emotions (β = -0.276), their indirect effect through mobile phone addiction (β = -0.280) was the most pronounced, even exceeding the direct effect.

The direct effect of family factors on negative emotions is weakly negative in urban males (β = -0.057), urban females (β = -0.02), and rural males (β = -0.022), while it is slightly positive in rural females (β = 0.147). In contrast, the indirect effect demonstrates a weak negative influence across all groups: urban males (β = -0.073), urban females (β = -0.103), rural males (β = -0.124), and rural females (β = -0.039).

Regarding exercise intensity, the direct effect on negative emotions is negative in urban males (β = -0.138) but positive in urban females (β = 0.120), rural males (β = 0.034), and rural females (β = 0.073). The indirect effects are weakly negative across all groups: urban males (β = -0.041), urban females (β = -0.169), rural males (β = -0.020), and rural females (β = -0.045).

## 4 Discussion

The present study employs a questionnaire survey of 493 youths to investigate the relationship between mobile phone addiction and negative emotions among youths. The analysis reveals the intricate mechanisms underlying these relationships and the influence of group differences. In light of the findings from the data analysis, this paper proposes that an understanding of the impact of mobile phone addiction on youth mental health offers a novel perspective and provides a scientific foundation for the development of effective interventions.

### 4.1 Differential explanations for mobile phone addiction

The study found notable disparities in mobile phone addiction between urban males and rural males, as well as between rural males and rural females. This finding aligns with previous research that has identified gender and regional differences in mobile phone addiction (70, 71). For instance, Rudolf & Kim (32) found that mobile phone addiction was more prevalent among females than males through a longitudinal investigation of the relationship between mobile phone addiction, gender, and mental health in Korean adolescents (32). Mobile phone addiction is higher among urban men males rural males, which may be due to the fact that urban males usually have higher socioeconomic status and education level, and consequently have greater access to smartphones and the Internet, thus increasing the risk of mobile phone addiction (72). Given these factors, the observed disparity in mobile phone addiction between urban and rural males may be attributed to differences in family economics and parental literacy levels between the two groups. In contrast, rural females have significantly greater mobile phone addiction than rural males, possibly because rural females are more restricted in terms of social interactions and social networks, which leads to a greater tendency to use their mobile phones for social interactions, thus increasing the risk of mobile phone addiction. Kosola’s (2024) study also noted the prevalence of social media addiction among adolescent females (73). Additionally, research has suggested that male addiction to mobile phones is often externally motivated, while female addiction tends to be driven more by internal factors such as enjoyment and emotional regulation (47). This implies that variations in the motivations behind mobile phone use could also play a significant role in explaining differences in mobile phone addiction between genders.

### 4.2 Differential effects of mobile phone addiction on negative emotions

The results from the RCS analysis revealed a statistically significant linear relationship between mobile phone addiction and negative emotions among urban male, rural male, and rural female adolescents. However, this relationship was not observed in urban female, adolescents whose mobile phone addiction had a relatively weak impact on their negative emotions. The reasons for this discrepancy may be more complex. On the one hand, the family environment and social support systems can exert a significant influence on the manner in which individuals cope with negative emotions and addictive behaviors (74, 75). To illustrate, Choi et al. (76) found that overall social support was associated with a 55% reduction in the risk of developing depression by longitudinally investigating perceived social support and depressive symptoms in 69,066 participants in the early years of COVID-19 (76). This comprehensive study favorably demonstrates the important role of social support in reducing the risk of depression. Additionally, Pei et al. (77) demonstrated from a brain neuroscience perspective that neural features of social support attenuated negative emotions (77)。 Conversely, there are discrepancies in the capacity of men and women to regulate their emotions when confronted with negative experiences (78). To illustrate, Zhang (2020) revealed that the mediating effect of cognitive reappraisal on the relationship between tolerance and depression among female adolescents was significantly greater than that observed among male adolescents (79). It is therefore plausible to suggest that urban females may be less susceptible to the negative effects of mobile phone addiction as a result of having greater access to social support and more effective emotion regulation strategies.

### 4.3 Mechanisms of mobile phone addiction on negative emotions

To elucidate further the discrepancies in the impact of mobile phone addiction on negative affect, we found that mobile phone addiction has a significant positive effect on negative emotions among urban males, rural males, and rural females through SEM. However, this effect was not statistically significant among urban females. This finding is similar to that of the RCS, and the consistency of results between the two methods serves to reinforce our confidence in these group difference conclusions.

Additionally, the direct effect of mobile phone addiction on negative emotions was more pronounced in the rural sample. Previous research has shown that family economic status and parental educational attainment are significant predictors of adolescent mobile phone addiction (80, 81). Adolescents from less affluent backgrounds are also more prone to experience negative emotional states (82–84). As previously indicated by Kim (2022), family economic distress has been demonstrated to precipitate an increase in anxiety and depression among adolescents (85). More seriously, a meta-analysis of the relationship between socioeconomic status and child psychopathology in the United States demonstrated that children from lower socioeconomic backgrounds are more likely to manifest psychopathological symptoms (86). In contrast, urban adolescents, typically from more affluent backgrounds exhibit greater psychological resilience (87), which contributes to a reduction in negative emotions. Our findings corroborate this pattern, showing that the socioeconomic status and the psychological resilience of urban adolescents has a stronger negative impact on negative emotions. This finding aligns with the conclusions of the study by Yuan et al. (88), which indicated a significant negative correlation between socioeconomic status and adverse emotional states, including stress, anxiety, and depression (88). This suggests that rural adolescents from economically disadvantaged families may experience more pronounced negative emotions when mobile phone addiction occurs.

Moreover, among adolescents residing in the same area, mobile phone addiction had a more pronounced direct positive effect on negative emotional states in males compared to females. On the one hand, there are differences in the psychological needs and behavioral patterns of males and females when coping with mobile phone use (89, 90). Although male adolescents may not display as much addictive behavior in terms of time spent on mobile phone use as females, they may be more susceptible to negative emotions in certain aspects of mobile phone addiction, such as gaming or social media (91). Significant gender differences have been observed in mobile phone addiction, with men displaying a greater proclivity for internet and gaming addiction (92, 93). Concurrently, the findings of a longitudinal study of internet gaming disorders (IGD) in adolescents by gender and educational stage revealed that the highest coefficients between IGD and anxiety symptoms were found in male adolescents aged 13–15 years old (94). Another study examined gender differences in the motivations behind mobile phone use (95). Males were more likely to engage with stimulating mobile games, while females tended to use mobile phones for emotional expression through communication (96, 97). This may render the males more susceptible to negative emotional states such as anxiety in the context of mobile phone addiction. On the other hand, previous studies indicates that emotion regulation is associated with mental health and there are gender differences (98). Males may be relatively inhibited in emotional expression, while females are better at expressing emotions (99). Delhom (2021) verified that females are better at emotion regulation than males (100). In this regard, women’s greater ability to regulate emotions may channel the negative emotions generated by mobile phone addiction through emotional expression. Again, this needs to be validated by further subsequent studies. Meanwhile, extrapolating from our other finding that women’s psychological resilience has a more significant reducing effect on negative emotions. Combining the two findings, it is reasonable to hypothesize that female psychological resilience has a key role in reducing negative emotions generated by mobile phone addiction.

In all four adolescent groups, psychological resilience had a negative effect on both mobile phone addiction and negative emotions, consistent with the findings of previous studies (101, 102). Psychological resilience not only reduces negative emotions directly, but also indirectly by inhibiting mobile phone addiction (103, 104). This is consistent with Shang’s suggestion that psychological resilience can alleviate the stress associated with mobile phone addiction (105). This highlights the potential of enhancing adolescents’ psychological resilience as an effective strategy to reduce the risk of mobile phone addiction and negative emotions. Additionally, the study determined that family factors and physical activity positively influenced psychological resilience across the different adolescent groups. This aligns with prior research indicating that physical activity enhances psychological resilience in Chinese college students (106). Another study systematically examined the relationship between adolescents’ psychological resilience and parental attitudes. The results of this study indicated that parental support and care increased adolescents’ psychological resilience (107). Furthermore, some studies have emphasized the positive impact of social support on adolescents’ psychological resilience (108, 109). Thus, adolescents may improve their psychological resilience through exercise, thereby indirectly reducing the risk of mobile phone addiction and negative emotions. Also, family and social support are crucial for them to reduce mobile phone addiction and cope with negative emotions.

In summary, the study aims to enhance our understanding of the relationship between mobile phone addiction and negative emotions, with a particular focus on how disparities in mobile phone addiction influence negative emotions across urban-rural and gender-based differences. This research is significant as it provides valuable insights into how mobile phone addiction interacts with negative emotions within varying socioeconomic contexts. Furthermore, it highlights the role of factors such as socioeconomic status, gender role identity, and family culture in shaping the mechanisms through which mobile phone addiction affects negative emotions. This multidimensional approach helps uncover the complex ways in which mobile phone addiction impacts adolescents’ emotional well-being across different demographic groups.

### 4.4 Limitations and future prospects

It should be noted that this study is not without limitations. Firstly, it should be noted that this study was a cross-sectional survey of adolescents (aged 17-22 years), which limits the generalizability of the findings to different age groups. Subsequent research surveys will be expanded to encompass a more diverse age range, including a representative sample of different age groups, thereby enhancing the generalizability of the findings. Furthermore, the survey was conducted in 2024, which precludes the possibility of discerning the trajectory of individual mobile phone addiction and negative emotions over time. It is not possible to discount the potential impact of differing time periods on the results. Subsequent studies will employ longitudinal surveys, following respondents over an extended period, thus enhancing the reliability of the results. Secondly, the cross-sectional nature of the survey precludes the possibility of establishing causality. It is reasonable to hypothesize that the causal pathways whereby mobile phone addiction leads to negative emotions and that negative emotions can exacerbate mobile phone addiction are both plausible. Further confirmation of this causal relationship is required through future longitudinal surveys. Furthermore, the impact of mobile phone addiction disparities on negative emotions was examined, with particular attention paid to the potential moderating influence of psychological resilience, physical activity, and family factors. These moderating roles of auxiliary variables do not negate the possibility that these variables may exert a dominant influence on the effects of negative emotions. Subsequent studies will further investigate the impact of these variables on negative affect.

## 5 Conclusion

In this study, a questionnaire was administered on 493 adolescents in Jiangxi Province to investigate the effects of mobile phone addiction on negative emotions, with a particular focus on differences across urban and rural areas as well as between genders. To analyze these relationships, a combination of correlation analysis, RCS analysis, and SEM was employed.

The findings of the study indicated that mobile phone addiction was significantly and positively correlated with negative emotional states among adolescents from both urban and rural backgrounds, as well as across gender groups. In contrast, psychological resilience was significantly and negatively associated with negative emotions, suggesting that higher resilience is linked to lower levels of negative emotional states. The study further revealed that rural adolescents were at a greater risk of experiencing negative emotions related to mobile phone addiction compared to their urban counterparts. Additionally, male adolescents exhibited a stronger tendency to develop negative emotional states due to mobile phone addiction than their female counterparts in the same place of residence. Among urban females, psychological resilience had the most pronounced negative impact on negative emotions, indicating that resilience plays a key role in mitigating the effects of mobile phone addiction in this group. In contrast, rural males showed the least significant negative effect of psychological resilience on their emotional states. The study also found that family factors and physical activity were positive contributors to psychological resilience. In conclusion, the findings underscore the importance of enhancing adolescents’ psychological resilience as an effective strategy for mitigating mobile phone addiction and its associated negative emotional impacts. Additionally, the study highlights the vital roles of physical activity and family support in fostering psychological resilience.

## Data Availability

The raw data supporting the conclusions of this article will be made available by the authors, without undue reservation.
